# Impact of systemic treatments on the course of HLA-B27-associated uveitis: A retrospective study of 101 patients

**DOI:** 10.1371/journal.pone.0230560

**Published:** 2020-03-31

**Authors:** Nabil Bouzid, Yvan Jamilloux, Roland Chapurlat, Pierre Pradat, Audrey De Parisot, Laurent Kodjikian, Pascal Sève

**Affiliations:** 1 Service de Médecine Interne, Hôpital de la Croix-Rousse, Hospices Civils de Lyon, Université Claude Bernard-Lyon 1, Lyon, France; 2 Service de Rhumatologie, Hôpital Édouard Herriot, Hospices Civils de Lyon, INSERM UMR 1033, Université de Lyon, Lyon, France; 3 Centre de Recherche Clinique, Hôpital de la Croix-Rousse, Hospices Civils de Lyon, Université Claude Bernard-Lyon 1, Lyon, France; 4 Service d’Ophtalmologie, Hôpital de la Croix-Rousse, Hospices Civils de Lyon, Université Claude Bernard-Lyon 1, Lyon, France; Oregon Health and Science University, UNITED STATES

## Abstract

**Purpose:**

To investigate the efficacy and tolerance of systemic treatments for the prevention of HLA-B27-associated acute uveitis (AU) recurrence.

**Methods:**

Retrospective review of patients with HLA-B27-associated uveitis followed in our tertiary center over a 15-year period. Systemic treatments were prescribed to patients with frequent (more than 2 flares per year) or severe uveitis, according to a step-up strategy

**Results:**

101 patients (51.5% of men, 88.1% of white Europeans) with a median age of 37 years. AU was mostly recurrent (68.3%) and associated with spondyloarthritis (60.4%). After a median follow-up duration of 22 months (3–73), 37.6% of the patients have received systemic treatment. 88.5% of the patients have been treated with sulfasalazine (SSZ) for ophthalmologic purposes (23/26). Methotrexate (MTX) and anti-TNFα agents have been initiated for a rheumatologic indication in 81.8% (9/11) and 100% of the patients (13/13), respectively. The annual uveitis relapse rate significantly decreased on SSZ (0.37 recurrences/year versus baseline 2.46 recurrences/year; p<0.001) and MTX (1.54 recurrences/year versus 4.17/year; p = 0.008). Patients under ADA for ophthalmologic purposes (n = 2) did not experience any recurrence.

**Conclusion:**

We report an open-label strategy to prevent the recurrences of HLA-B27-associated AU. First-line sulfasalazine reduced uveitis relapses. The use of anti-TNFα agents for ophthalmologic purposes was unnecessary with rare exceptions.

## Introduction

Acute uveitis (AU) associated with the Human Leukocyte Antigen B27 (HLA-B27) is the most frequent cause of uveitis [1,2]. HLA-B27-associated uveitis may occur as an isolated eye disease, but is commonly associated with spondyloarthritis. The combination of uveitis and HLA-B27 has been described since 1973 and concomitantly with ankylosing spondylitis [3,4]. In white European population, the prevalence of HLA-B27 is 7% and reaches 80% in patients with spondyloarthritis [5]. Ocular involvement in spondyloarthritis is the most common extra-articular manifestation of the disease, affecting approximately 30% of the patients [6,7]. Among these ophthalmologic manifestations, acute anterior uveitis is the most common, especially in HLA-B27 positive patients with ankylosing spondylitis [6,8].

The prognosis of HLA-B27-associated uveitis is usually good in the long term but complications and recurrences may occur. The most common complications are posterior synechiae (13–90%) [9] and cataract in young adults (7–28%) [8]. Ocular hypertension (8–20%), papillitis (2–18%) and cystoid macular edema (6–13%) are less frequent [6]. The frequency of relapses varies from 0.6 to 3.3 flares / year according to studies and decreases over time [8].

In the majority of AU, topical corticosteroids, or in more severe cases periocular corticosteroids, associated with cycloplegic eye drops are sufficient to resolve inflammation [10–12]. However, recurrences may lead to start a disease-modifying anti-rheumatic drug (DMARD), such as sulfasalazine (SSZ) and methotrexate (MTX), as well as anti-TNFα agents [13]. These agents are used in case of sight-threatening complications (macular edema), in case of ocular side-effects (e.g., steroid-induced glaucoma), or in relapsing diseases. The rheumatic disease is also considered when introducing a systemic treatment for uveitis.

A reduction in the recurrence rate of AU has been reported with SSZ, MTX and anti-TNFα agents, especially infliximab (IFX) and adalimumab (ADA) [14–23]. However, studies investigating the effects of systemic treatments on the course of HLA-B27-associated uveitis are rare. The main aim of this study was to evaluate an open-label step-up strategy for the prevention of recurrences of HLA-B27-associated AU. The secondary aim was to describe the efficacy and tolerance of systemic treatments for the prevention of recurrences and the visual prognosis.

## Patients and methods

### Study design and population

This is a retrospective analysis of the medical records of patients with HLA-B27-associated uveitis, with at least one episode of AU, referred to the Department of Internal Medicine (Hôpital de la Croix-Rousse, Hospices Civils de Lyon, Lyon, France) between January 2003 and April 2018. Patients were referred either by the Department of Ophthalmology (Hôpital de la Croix-Rousse, Hospices Civils de Lyon, Lyon, France) or by ophthalmologists working in urban health service. Exclusion criteria were uveitis associated with another etiology than HLA-B27.

To avoid missing data, a standardized survey was sent to the ophthalmologist or to the general practitioner and a telephone interview with the patient was scheduled, and the data were cross-checked from the patient's medical record. The study design complies with French law. The institutional ethics committee of the Hospices Civils de Lyon approved the study. All participants were orally informed of the study and the participation to the study was notified in the patient’s medical record.

**Fig 1:**
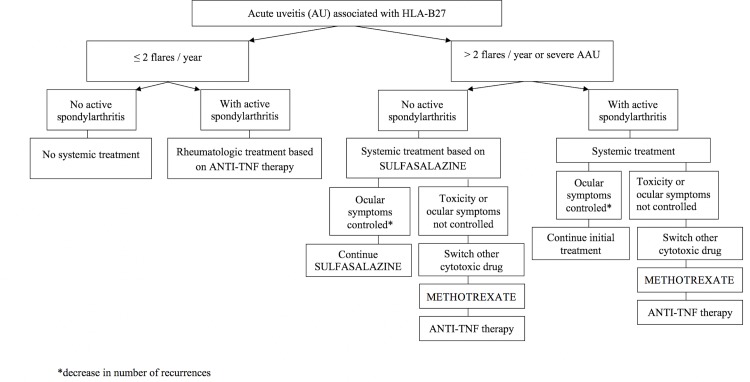
Algorithm to treatment of HLA-B27 associated uveitis. *decrease in number of recurrences.

The systemic treatment was initiated by the internist or the rheumatologist, according to ophthalmologic data, rheumatologic data and clinical characteristics of patients, including the number and severity of relapses. All patients were questioned about back pain, psoriasis, enthesopathy, and bowel symptoms. The severity of uveitis includes prognostic factors, for visual loss such as: posterior uveitis, macular edema, uveitic complications of glaucoma, advanced cataract, synechiae, severe band keratopathy or ocular hypotony [36]. All patients were treated with the same step-up therapeutic strategy and by the same practitioner (PS) according to the algorithm shown in **Fig 1**. As described in previous studies, no systemic treatment was initiated for non-recurrent and/or non-severe uveitis [13–23]. Patients with active spondyloarthritis were treated by the rheumatologist or by the internist according to the ASAS-EULAR management recommendations for spondyloarthritis [24]. Patients with recurrent uveitis (more than 2 /year) or severe uveitis were referred for systemic therapy [15].

SSZ was administered orally every day, with an initial dosage of 500 mg/day and up to 2000 mg/day. MTX was administered, once a week, orally or subcutaneously (10–25 mg / week), with folic acid (5 mg) 24 to 48 hours later. Among anti-TNFα agents, ADA was administered every two weeks, subcutaneously (40mg/2 weeks), IFX was administered intravenously (5mg/kg at baseline, week 2, week 6 and every 8 weeks thereafter), etanercept (ETA) was administered once a week, subcutaneously (50 mg/week).

A dose adjustment was performed by the practitioner who initiated the treatment (internist or rheumatologist), based on efficacy and tolerance. When patients were stable for several consecutive visits, the dose was gradually reduced over time. The treatment was usually stopped in case of toxicity.

### Clinical examinations and investigations

Each patient report was retrospectively reviewed for demographic data (age at first visit, sex, ethnic group), ophthalmologic characteristics (laterality, recurrences, associated ophthalmic complications including posterior synechiae, secondary cataract, ocular hypertension, papillitis, and macular edema), and for the presence of an associated spondyloarthritis using the European Spondylarthropathy Study Group Criteria [25] and the ASAS criteria for spondyloarthritis [26, 27].

Diagnoses of recurrence were established by ophthalmologists during follow-up, using the SUN international criteria [28] for the anatomical classification of uveitis. At each recurrence, all patients had a complete ophthalmologic examination, including a slit-lamp examination and an indirect ophthalmoscopy.

The treatment efficacy was determined by the uveitis relapse rate as defined by the number of AU flares/patient/year, and the presence of sight-threatening complications.

### Statistical analysis

Data are described as frequencies and percentages for categorical variables, and medians (25th–75th percentiles) for quantitative variables. Categorical variables were compared using the Chi2 test or Fisher’s exact test, as appropriate. Relapse rates were compared using a Poisson regression model. All tests were two-sided and statistical significance was set at p<0.05. All statistical analyses were performed using R v.3.5.2 (R Foundation for Statistical Computing, Vienna, Austria).

## Results

### Population

During the study, 101 patients with HLA-B27-associated uveitis were referred to our Department of Internal Medicine. Patients’ clinical characteristics are presented in **Table 1**.

**Table 1:** Epidemiologic and ophthalmologic characteristics of patients with HLA-B27-associated uveitis.

Characteristic		Patients (N = 101)
Gender	Female	49 (48.5)
	Male	52 (51.5)
Median age (IQR)		37 (26–47)
Ethnicity	White European	89 (88.1)
	North-African	5 (4.9)
	Central-African	5 (4.9)
	Caribbean	1 (1)
	South-American	1 (1)
Rheumatologic history	Ankylosing spondylitis	55 (54.4)
	Reactive arthritis	3 (3)
	Psoriatic arthritis	2 (2)
	Inflammatory bowel disease	1 (1)
	No inflammatory rheumatism	40 (39.6)
Ophthalmologic manifestations	Anterior	95 (94.1)
	Vitreous haze	9 (8.9)
	Cystoid Macular Edema	7 (6.9)
	Papillitis	6 (5.9)
	Vasculitis	2 (2)
	Chronic form	10 (9.9)
	Bilateral involvement	10 (9.9)
	Granulomatous uveitis	3 (3)
	Elevated IOP	6 (5.9)

All data shown are n (%), unless otherwise specified.

IOP, intraocular pressure; IQR, interquartile range

The median age at uveitis onset was 37 years (interquartile range 26–47) with a sex ratio close to 1 (men = 52, women = 49). The patients were mostly white Europeans (88.1%). Sixty-one patients (60.4%) presented with spondyloarthritis, including ankylosing spondylitis (n = 55, 90.2%), psoriatic arthritis (n = 2), reactive arthritis (n = 3), and spondyloarthritis associated with inflammatory bowel disease (n = 1). Among these patients, the rheumatic involvement preceded uveitis in 46% of the cases (n = 28). In contrast, patients had initially isolated ophthalmologic manifestations in 54% of the cases (n = 33). Patients reported chronic low back pain (n = 42, 68.8%), unilateral or bilateral buttock pain (n = 13, 21.3%), alternating buttock pain (n = 11, 18%), asymmetric oligoarthritis (n = 8, 13.1%), enthesitis (n = 5, 8.2%), and dactylitis (n = 1, 1.6%).

The uveitis was anterior (n = 95, 94.1%), or complicated by macular edema (n = 7, 6.9%), papillitis (n = 6, 5.9%) and vasculitis (n = 2, 2%). Overall, uveitis was more frequently acute (n = 91, 90.1%), unilateral (n = 91, 90.1%), non-granulomatous (n = 98, 97%), and non-hypertensive (n = 85, 84.1%). Patients with granulomatous uveitis had psoriatic arthritis (n = 2, 66.7%) or reactive arthritis (n = 1, 33.3%). The main complication of AU was the presence of synechiae (n = 33, 32.6%). Six patients (6%) had a hypopyon. Anterior uveitis was recurrent in 69 patients (68.3%).

All patients received topical corticosteroids and cycloplegic eye drops and, in selected cases, periocular corticosteroids, for AU recurrences.

Thirty-eight patients (37.6%) received a systemic treatment (**Table 2**). Patients were treated with SSZ (n = 26, 68.4%), MTX (n = 11, 28.9%) and anti-TNFα agents (n = 13, 34.2%); because of recurrent and/or severe uveitis (n = 22, 57.9%), inflammatory rheumatism (n = 11, 28.9%) or for both (n = 5, 13.2%). Among anti-TNFα agents, ADA was introduced in 6 patients (46.1%), ETA in 5 patients (38.5%) and IFX in 2 patients (15.4%).

**Table 2:** Systemic treatments of patients with HLA-B27-associated uveitis.

Systemic therapy	Patients (n = 38)	For ophthalmologic conditions	For rheumatologic conditions	For both conditions
Sulfasalazine	26 (68.4)	21	3	2
Methotrexate	11 (28.9)	2	5	4
Adalimumab	6 (15.8)	0	4	2
Etanercept	5 (13.2)	0	5	0
Infliximab	2 (5.2)	0	2	0

Patients could have been treated with more than one treatment.

All data shown are n (%).

**Fig 2:**
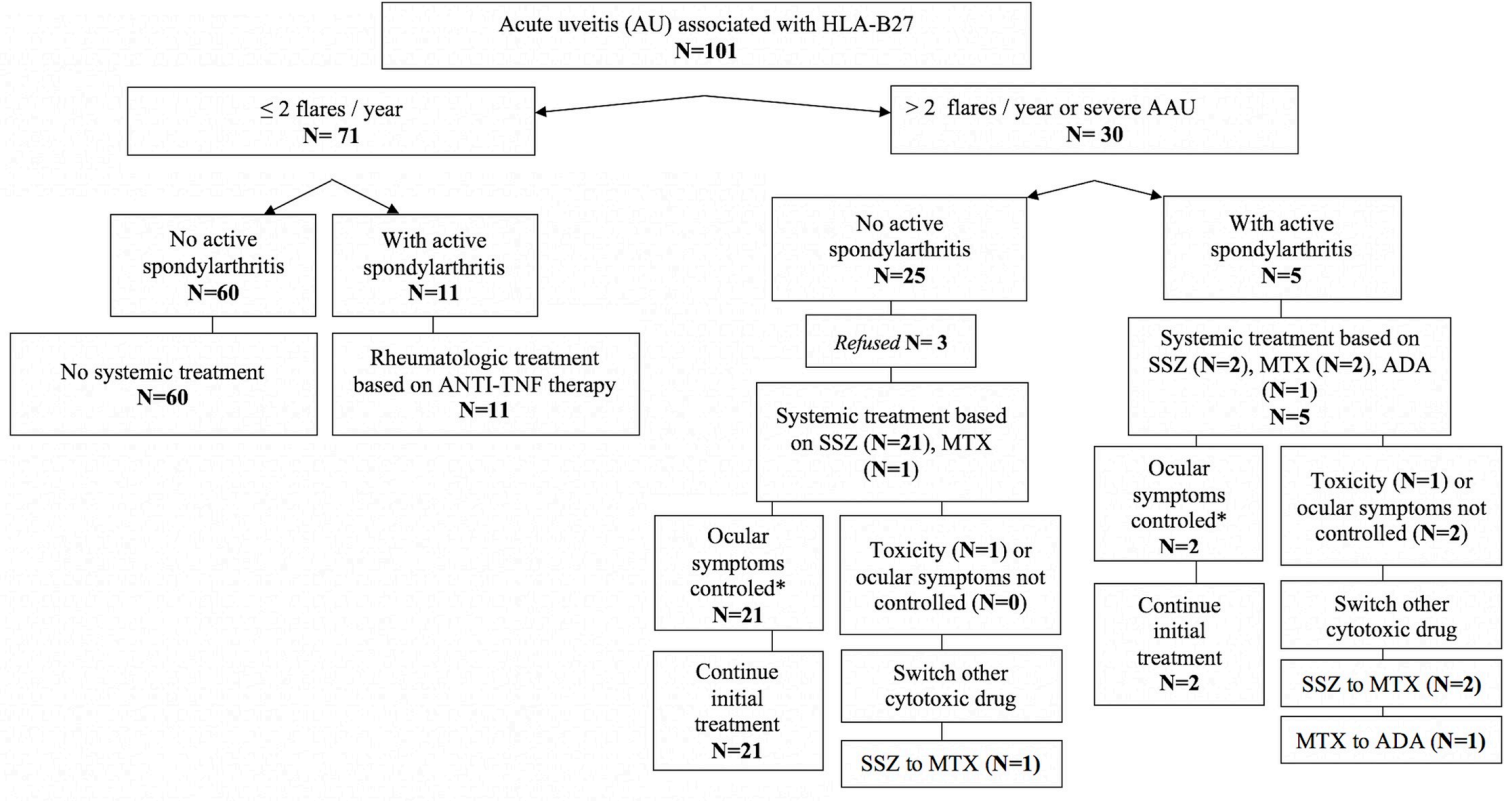
Flowchart to treatment of HLA-B27 associated uveitis.

SSZ was prescribed for ophthalmic manifestations in 88.5% of the cases (n = 23). Three (11.5%) patients had SSZ for rheumatologic purposes only. MTX was started for rheumatologic purposes in 81.8% of the cases (n = 9) and for ophthalmic manifestations in 54.5% (n = 6). Two (18.2%) patients had MTX for ophthalmologic purposes only. Regarding anti-TNFα agents, ADA was started for rheumatologic purposes in 100% of the cases but also for ophthalmic manifestations in 2 of the 6 patients. ETA (n = 5, 13.2%) and IFX (n = 2, 5.2%) were always started for rheumatologic purposes. No anti-TNFα agent was started for an ophthalmologic involvement only. The flowchart shows the distribution and the modifications of the different treatments (**Fig 2**). The median follow-up duration in our study was 22 months (3–73). Sixty-three (62.4%) patients were followed for more than one year.

### Treatment efficacy in patients with less than two recurrences per year

#### Patients without systemic therapy

Sixty patients (59.4%) had at least one episode of AU but did not require any systemic therapy. These patients had significantly lower relapse rates (0.87 flares/ year) than patients treated for uveitis recurrences (2.57 flares/ year, p<0.001). No AU relapse occurred during follow-up in 39.7% of the patients (n = 25). Four patients (6.7%) without systemic therapy developed uveitis complications (papillitis (n = 1) and macular edema (n = 3)) which did not require the introduction of a systemic treatment.

#### Patients with DMARDs and anti-TNFα agents for rheumatologic purposes

Eleven patients (10.9%) had at least one episode of uveitis but the treatment was introduced for rheumatologic purposes. Initially, patients with spondyloarthritis were treated with SSZ (n = 3, 27.3%), MTX (n = 3, 27.3%), ADA (n = 2, 18.2%), ETA (n = 3, 27.3%). All patients treated with SSZ had to switch their treatment to MTX (n = 2, 66.7%) and ETA (n = 1, 33.3%) because of gastric toxicity (n = 1) and insufficient rheumatologic response (n = 1). One patient treated with MTX had to switch to ETA because of hematologic toxicity. Due to inefficacy, two patients treated with ETA had to switch to ADA and two patients treated with MTX had to switch to IFX. The average number of uveitis recurrences was 1.29 flares/year before treatment and 0.20 flares/year after (p<0.001). No patient treated with SSZ (n = 3, 27.3%) and IFX (n = 2, 18.2%) for rheumatologic conditions had uveitis recurrences. The average recurrence rate under MTX, ADA and ETA were respectively 0.20, 0.23 and 0.13 flares/year.

#### Treatment efficacy in patients with uveitis recurrences

Thirty patients (29.7%) with recurrent or severe AU required a systemic treatment. Among these patients, 5 (16.7%) also had active inflammatory rheumatism. In patients with ophthalmologic manifestations only, 3 (12%) declined SSZ (**Fig 2**).

The number of uveitis flares before treatment was 2.46 flares/year in patients who received SSZ; 4.17 flares/year in patients who received MTX and 2.17 flares/year in patients who received ADA.

**Fig 3:**
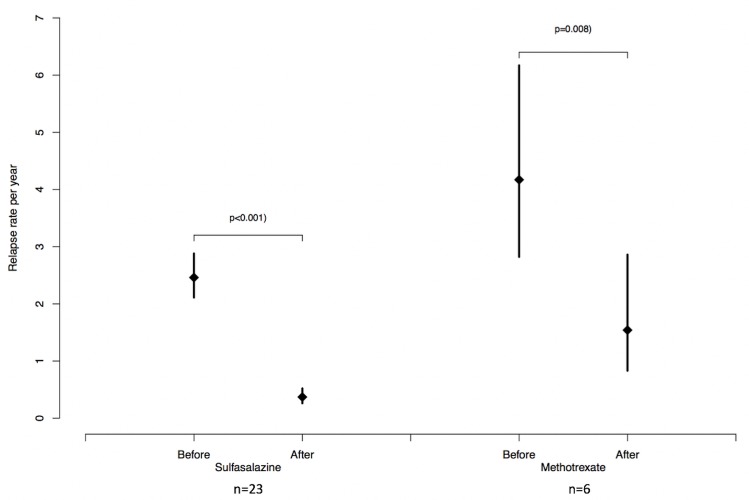
Uveitis relapse rate per year in patients with uveitis recurrences treated with sulfasalazine or methotrexate.

During follow-up, the AU relapse rates were significantly reduced in patients receiving either systemic SSZ, MTX or anti-TNFα agents. Patients treated with SSZ developed 0.37 AU flares/year (p<0.001 compared to pre-treatment values). Patients treated with MTX showed a significant reduction in the AU relapse rate from 4.17 before treatment to 1.54 flares/year (p = 0.008) (**Fig 3**). The patients treated with ADA (n = 2) did not develop AU during follow-up.

In patients treated for ophthalmologic purposes, 3 patients on SSZ switched for MTX (1 treatment failure, 2 toxicities) and 1 patient under MTX switched for adalimumab (ADA) due to lack of efficacy.

No AU relapse occurred during follow-up in 9 patients (39.1%) treated with SSZ, in 3 (50%) patients treated with MTX and in 2 (100%) patients treated with ADA.

During follow-up, papillitis occurred in 2 (8.7%) patients and was treated with SSZ. No ocular complications were reported under MTX and ADA.

#### Tolerance

Eight of 26 patients (30.8%) treated with SSZ, 5/11 (45.4%) patients treated with MTX, 1/5 (20%) patient treated with ETA and 1/2 (50%) patient treated with IFX experienced adverse events (**Table 3**). The proportion of patients with adverse events did not differ significantly according to systemic treatments (p = 0.330). Four serious adverse events led to treatment discontinuation in patients treated with SSZ (50%), 3 with MTX (60%), 1 with ETA (100%) and 1 with IFX (100%) respectively.

**Table 3:** Adverse events in patients with HLA-B27-associated uveitis receiving systemic treatments.

Adverse events	SSZ (n = 26)	MTX (n = 11)	ADA (n = 6)	ETA (n = 5)	IFX (n = 2)
Any adverse event**	8 (30.8)	5 (45.4)	0 (0)	1 (20)	1 (50)
**Adverse event leading to discontinuation**	4 (15.4)	3 (27.3)	0 (0)	1 (20)	1 (50)
Cardiac rhythm disorder	1* (3.8)	0	0	0	0
Infectious pneumonia	1* (3.8)	0	0	0	0
Hypersensitivity pneumonitis	0	1* (9.1)	0	0	0
Drug eruption	2* (7.7)	0	0	0	0
Repeated episcleritis	0	0	0	1* (20)	0
Allergic reaction	0	1* (9.1)	0	0	0
Anaphylactic shock	0	0	0	0	1* (50)
Cytopenia	0	1* (9.1)	0	0	0
**Adverse event leading to death**	0	0	0	0	0

All data shown are n (%).

*Adverse event leading to discontinuation of the treatment

**Others adverse events include gastrointestinal disorders, dyspepsia, epigastric pain

#### Status at final follow-up

At final follow-up, 30.7% of the patients had a systemic treatment. 16 patients were treated with SSZ; for ophthalmologic purposes in 100% (16/16). 4 patients were treated with MTX for ophthalmologic purposes in 50% (2/4). 11 patients were treated with anti-TNFα agents for a rheumatologic involvement (ADA (n = 6), IFX (n = 2) and ETA (n = 3)).

The long-term prognosis of patients was globally favourable. Seven patients only (6.9%) had uveitis sequelae with synechiae and one had active uveitis (1%). All patients had a normal intraocular pressure (IOP) at the end of follow-up. Ten patients underwent surgery during follow-up including surgical synechialysis (n = 1), cataract surgery (n = 10), epiretinal membrane surgery (n = 1) and retinal detachment surgery (n = 1). No patient needed filtration surgery.

## Discussion

Studies investigating the effects of systemic treatments on the course of HLA-B27-associated uveitis are rare [14–23]. We describe the efficacy and safety of systemic treatments following an step-up open-label strategy in patients with HLA-B27-associated uveitis. Our study suggests that SSZ positively influences the course of HLA-B27-associated uveitis and that our strategy may reduce relapses with mild side-effects. No patient received anti-TNFα agents for ophthalmologic purposes only.

Therefore, we applied the same therapeutic strategy to patients who received treatments according to their rheumatologic and ophthalmologic characteristics. For comparison, we divided patients into 3 groups: patients who did not require a systemic treatment, those who needed DMARDs or anti-TNFα agents for rheumatologic purposes, and those who needed a systemic treatment for ophthalmologic purposes with or without a rheumatologic indication.

Patients treated with SSZ showed a significant reduction in the AU relapse rate from 2.46 before treatment to 0.37 flares/year, with a relative risk reduction of 85%. Dougados et al., then Munoz-Fernandez et al. in a small prospective longitudinal open-label study (n = 10), reported a similar decrease in recurrence rate on SSZ [29,15]. Similarly, the criterion for a systemic treatment was 3 or more episodes of AU the year before inclusion or 2 or more episodes of uveitis 3 months before the study. Benitez et al. also showed SSZ efficacy in a prospective randomized study (versus placebo) including 22 patients with HLA-B27 [14]. A recent study investigating the effects of SSZ and MTX on the course of HLA-B27-positive remitting acute uveitis reported a significant reduction in uveitis rates in both groups [30]. SSZ with its mild side-effects (rash and digestive disorders), and its low cost, should be a first-line treatment in patients with recurrent and severe episodes of uveitis. However, clinicians should keep in mind that rare severe cytopenias and skin reactions have been described [31]. As in the literature, one third of our patients experienced adverse effects that led to SSZ discontinuation in one sixth of the patients. Three patients requiring the introduction of systemic therapy refused SSZ. Indeed, a daily oral administration may be restrictive for some patients. As rheumatologic effectiveness is restricted to peripheral symptoms, only 3 of our patients received SSZ for rheumatologic purposes [31].

Patients treated with MTX showed a significant reduction in the AU relapse rate from 4.17 before treatment to 1.54 flares/year, with a relative risk reduction of 63%. As MTX is teratogenic and might be associated with exceptional severe adverse events, such as bone marrow failure, lung damage and hepatotoxicity [32], this drug should be a second-line therapy in patients with failure or intolerance to SSZ. In 2008, an open clinical trial of 9 patients also suggested the efficacy of MTX in decreasing the recurrence rate of AU, in patients with more than 3 recurrences of AU the year before, at an initial dosage of 7.5 to 20 mg/week [16]. Meyer Zu Hoerste et al. recently reported that MTX reduced the uveitis relapse rate in 20 HLA-B27-positive AU patients and showed a beneficial effect on uveitis-related macular edema [30]. This treatment, which is efficient for moderate and severe peripheral involvement, was mostly prescribed in our study for rheumatologic purposes [33].

Our patients treated with ADA or ETA for rheumatologic purposes showed a lower AU relapse rate under treatment. Similarly, 2 patients treated with IFX for rheumatologic purposes did not experience any recurrence. Two patients treated for both ophthalmologic and rheumatologic manifestations with ADA did not develop any AU relapse during follow-up. Results should be interpreted cautiously because of the low number of patients. Anti-TNFα agents are effective in reducing uveitis recurrences by 50% to 80% according to studies. In 2008, a multicenter uncontrolled open-label study by Rudwaleit et al., demonstrated a more than 50% reduction in uveitis flares in patients with ADA [19]. Several observational studies and one meta-analysis showed that the soluble TNF receptor fusion protein (ETA) is less effective in preventing anterior uveitis, compared to the anti-TNFα monoclonal antibodies (IFX and ADA) [17,18,34]. However, the American and European rheumatology organizations do not recommend a treatment with these drugs over a treatment with ETA in patients with a single uveitis episode [35].

ADA and IFX are the biological agents of choice in the management of uveitis associated with spondyloarthritis according to recent studies [36]. In patients with recurrent or severe uveitis, anti-TNFα monoclonal antibodies should be preferred over ETA [35,37]. In the absence of a rheumatologic indication, SSZ and MTX could be considered as a priority in patients with severe and recurrent uveitis. In patients with axial rheumatic diseases, anti-TNFα agents should be preferred [24].

As in the literature, our study showed the efficacy of different treatments to prevent AU recurrences. In addition, our study parallels different treatments within a same step-up therapeutic strategy, with a large number of patients and a long follow-up time. Our study is one of the few studies evaluating the safety of systemic treatments for the prevention of HLA-B27-associated uveitis recurrences. Even if adverse events were not frequent in our patients, these treatments have short or long term adverse effects, especially anti-TNFα agents [38]. With regards to the visual prognosis, it was favourable in most patients with only 7% of long term complications (6.9% of synechiae and one case of chronic uveitis at final follow-up).

This study has limitations, because it is retrospective and therefore prone to patient memory bias and missing data, which is why data were cross-checked from the patient's medical record, and a standardized survey was sent to the ophthalmologist or to the general practitioner and a telephone interview was scheduled with the patient. Despite overlapping data, some records such as Tyndall values or visual acuity were often missing. Consequently, we focussed on recurrences and overall visual prognosis rather than visual acuity to evaluate treatment efficacy. Another limitation of the study is the regression to the mean. These results encourage the completion of a prospective study with a high-level of evidence. This study was not designed to address the superiority of a systemic treatment over the others but to suggest an effective therapeutic strategy with different treatment lines. In our opinion, a systemic therapy should be introduced for the prevention of HLA-B27-associated uveitis in patients with more than 2 relapses per year, or in case of severe uveitis. The introduction of a systemic treatment should be a multidisciplinary decision taking into account the underlying rheumatic disease and its characteristics (axial or peripheral).

In conclusion, most patients referred to our tertiary uveitis center for the management of HLA-B27-associated uveitis did not receive a systemic treatment. Due to its good therapeutic index, SSZ was used as a first-line therapy in patients with frequent recurrences of uveitis (more than 2 per year) without spondyloarthritis or with mild to moderate peripheral rheumatologic manifestations. In case of adverse events or contraindication to SSZ, MTX was used. The use of anti-TNFα agents for specific ophthalmologic purposes is unnecessary with rare exceptions. Their use seems restricted to patients with severe axial spondyloarthritis.

## Supporting information

S1 Data (XLSX)
